# Upscaling Mixing-Controlled Reactions in Unsaturated Porous Media

**DOI:** 10.1007/s11242-021-01710-2

**Published:** 2021-11-09

**Authors:** Lazaro J. Perez, Alexandre Puyguiraud, Juan J. Hidalgo, Joaquín Jiménez-Martínez, Rishi Parashar, Marco Dentz

**Affiliations:** 1 Division of Hydrologic Sciences, Desert Research Institute, Reno, NV USA; 2 Spanish National Research Council (IDAEA-CSIC), Barcelona, Spain; 3 Géosciences Rennes, UMR 6118, Université de Rennes 1, CNRS, 35042 Rennes, France; 4 Swiss Federal Institute of Aquatic Science and Technology, Eawag, Dübendorf Switzerland; 5 Department of Civil, Environmental and Geomatic Engineering, ETH Zurich, Zürich, Switzerland

**Keywords:** Upscaling, Mixing, Unsaturated porous media

## Abstract

We study mixing-controlled chemical reactions in unsaturated porous media from a pore-scale perspective. The spatial heterogeneity induced by the presence of two immiscible phases, here water and air, in the pore space generates complex flow patterns that dominate reactive mixing across scales. To assess the impact of different macroscopic saturation states (the fraction of pore volume occupied by water) on mixing-controlled chemical reactions, we consider a fast irreversible reaction between two initially segregated dissolved species that mix as one solution displaces the other in the heterogeneous flow field of the water phase. We use the pore-scale geometry and water distributions from the laboratory experiments reported by Jiménez-Martínez et al. (Geophys. Res. Lett. 42: 5316–5324, 2015). We analyze reactive mixing in three complementary ways. Firstly, we post-process experimentally observed spatially distributed concentration data; secondly, we perform numerical simulations of flow and reactive transport in the heterogeneous water phase, and thirdly, we use an upscaled mixing model. The first approach relies on an exact algebraic map between conservative and reactive species for an instantaneous irreversible bimolecular reaction that allows to estimate reactive mixing based on experimental conservative transport data. The second approach is based on reactive random walk particle tracking simulations in the numerically determined flow field in the water phase. The third approach uses a dispersive lamella approach that accounts for the impact of flow heterogeneity on mixing in terms of effective dispersion coefficients, which are estimated from both experimental data and numerical random walk particle tracking simulations. We observe a significant increase in reactive mixing for decreasing saturation, which is caused by the stronger heterogeneity of the water phase and thus of the flow field. This is consistently observed in the experimental data and the direct numerical simulations. The dispersive lamella model, parameterized by the effective interface width, provides robust estimates of the evolution of the product mass obtained from the experimental and numerical data.

## Introduction

In porous media, flow can vary and greatly influence the way dissolved substances are displaced. Complex flow regimes impact solute spreading and chemical reaction dynamics (Dentz et al. 2011), which are critical processes that need to be understood to develop accurate models, for example, in the remediation of groundwater contamination (Barros et al. 2011, 2013). Unsaturated porous media, where immiscible and partially miscible fluids coexist in the pore space, have a heterogeneous configuration of phases whose spatial and temporal rearrangement plays a key role in many natural and engineered processes from the biosphere to industrial applications (Fredlund and Houston 2009; Bouazza et al. 2013; Tan et al. 2016). As a natural example, in the unsaturated zone between the soil surface and groundwater table, a small change in the water saturation (fraction of the pore volume occupied by water) and in the distribution of the immiscible phase (i.e., air) modifies solute transport, mixing and chemical reactions (Padilla et al. 1999; Rood 2004; Mahmoodlu et al. 2016).

In saturated media, these processes are affected by medium heterogeneity (Dentz et al. 2011; de Anna et al. 2014b; Berkowitz et al. 2016; Valocchi et al. 2019). When two or more fluid phases are present, an additional level of complexity arises (De Gennes 1983). Laboratory experiments in bead packs (Matsubayashi et al. 1996), sand columns (Padilla et al. 1999; Bromly and Hinz 2004), and recently in milli- and microfluidics experiments (Jiménez-Martínez et al. 2015, 2017; Leontidis et al. 2020) have shown that the distribution of the immiscible phase combined with medium heterogeneity alters mixing and dispersion behaviors in the system compared to fluid saturated media. In fact, experimental and numerical studies have highlighted the saturation dependence of dispersion coefficients (Nützmann et al. 2002; Raoof and Hassanizadeh 2013; Leontidis et al. 2020), and the occurrence of non-Fickian transport (Bromly and Hinz 2004; Zoia et al. 2010; Aziz et al. 2018; Hasan et al. 2019, 2020). Furthermore, numerical simulations showed that the emergence of highly heterogeneous velocity distributions is related to the development of high-velocity regions (preferential flow paths) and low-velocity regions (stagnation zones) (Triadis et al. 2019; Velásquez-Parra et al. 2021), which leads to enhanced solute spreading and chemical reactivity (Sole-Mari and Fernàndez-Garcia 2018; Nissan and Berkowitz 2019; Jiménez-Martínez et al. 2020).

Large-scale transport descriptions in terms of saturation-dependent hydrodynamic dispersion coefficients or non-local models such as continuous time random walks and multirate trapping models describe the behavior of the average solute concentration. They characterize solute spreading rather than mixing (Dentz et al. 2011). The quantification of the impact of the medium structure and saturation state on mixing is key to understand the coupled transport and reaction behavior in unsaturated porous media. Jiménez-Martínez et al. (2015) and Jiménez-Martínez et al. (2020) studied the mechanisms of mixing and mixing-induced chemical reactions under different saturation conditions. These authors find increased solute mixing for decreasing saturation.

While these works provide invaluable insight concerning the mechanisms of solute dispersion and mixing under variable saturation conditions, the quantification of solute mixing and chemical reaction in a Darcy-scale continuum model remains an open question. Here, we probe the impact of the saturation state of a porous medium on reactive mixing for a fast irreversible bimolecular reaction and develop an upscaled continuum approach in terms of effective dispersion coefficients to quantify the pore scale mixing and reaction dynamics. To this end, we employ three complementary approaches using experimental data, numerical simulations, and an upscaled mixing model. The first approach uses an exact algebraic map between conservative and reactive species (Perez et al. 2020) in order to assess reactive mixing from experimental tracer data in porous media under variable saturation conditions (Jiménez-Martínez et al. 2015). The second approach is based on the hypothesis that the bulk of the experimental observations can be reproduced adopting simple, physically based rules of transport and reaction. To this end, the flow field in the water phase of the porous medium is determined numerically and reactive mixing is quantified through numerical reactive random walk particle tracking simulations. The third approach is based on the dispersive lamella model (Perez et al. 2019b, 2020; Puyguiraud et al. 2020) for the upscaling of the reactive mixing in saturated porous media. The approach decomposes the mixing front between two segregated constituents in material elements, so-called lamellae, which represent the Green function of the small-scale advection-diffusion problem. It is approximated by a Gaussian-shaped distribution, whose width is characterized by an effective dispersion coefficient that accounts for the impact of spatial heterogeneity on effective solute mixing (Dentz et al. 2000; Cirpka 2002; Jose and Cirpka 2004). This approach differs from other lamella-based reactive mixing models (de Anna et al. 2014a; Le Borgne et al. 2014; Bandopadhyay et al. 2017) that consider the lamellae as independent and therefore, mass transfer and superposition of lamellae is not accounted for. The dispersive lamella model, on the other hand, accounts intrinsically for lamella interactions and the action of flow deformation and transverse diffusion on the width of the mixing interface. Thus, the dispersive lamella captures the full evolution of mixing caused by fluid deformation at early times and dispersive mixing at late times within a single mathematical approach.

The paper is organized as follows. Section 2 describes the experimental data analysis, the reactive random walk particle tracking (RWPT) simulations and the upscaled dispersive lamella approach. Section 3 presents and discusses the numerical simulations and dispersive lamella approach versus experimental data. The impact of saturation degree on the evolution of the mixing interface, and reaction efficiency is studied using the numerical simulations and the dispersive lamella approach. Finally, Sect. 4 summarizes the main results and provides an outlook on reaction prediction in unsaturated porous media.

## Methodology

**Fig. 1:**
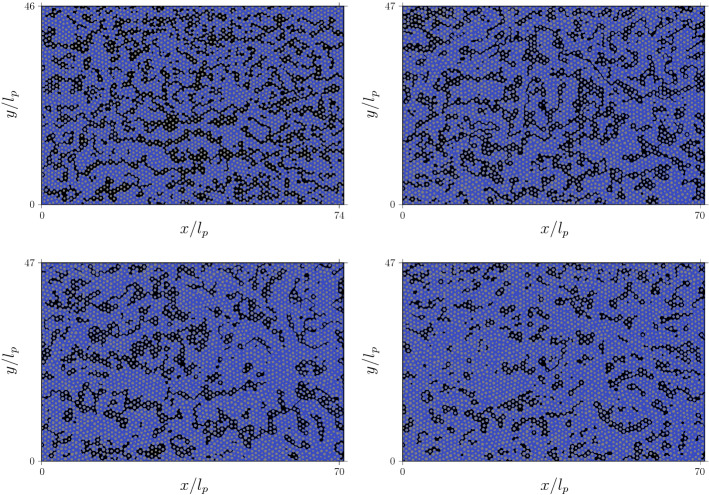
Pore geometries and saturation degrees for, top left $S_w = 0.70$ (scenario SW70) (Jiménez-Martínez et al. 2015), top right $S_w = 0.71$ (SW71), bottom left $S_w = 0.77$ (SW77), and bottom right $S_w = 0.83$ (SW83) (Jiménez-Martínez et al. 2017). The wetting fluid is shown in blue, the non-wetting fluid in black, and solid grains in gray. The scenario SW70 (top left) is also used for the validation of the numerical model

We study pore scale reactive mixing in the flow through the water phase of a partially saturated porous medium under different saturation conditions as illustrated in Fig. 1. We consider a displacement scenario in which a solution of the reactant species *A* displaces a solution of reactant species *B*. At the mixing interface between the two solutes, the species *C* is produced according to the instantaneous irreversible chemical reaction

1 $$\begin{aligned} A + B \rightarrow C \end{aligned}$$

The pore-scale reactive transport problem is described by the advection-diffusion reaction equation

2 $$\begin{aligned} \frac{\partial c_i(\mathbf {x},t)}{\partial t} + \mathbf {v(x)}\cdot \nabla c_i(\mathbf {x},t) - D \nabla ^2c_i(\mathbf {x},t) = r_i(\mathbf {x},t), \end{aligned}$$

where the reactants and product concentrations are $c_i(\mathbf {x},t)$ with $i = A,\,B,\,C$, the velocity vector at location $\mathbf {x}$ is denoted by $\mathbf {v(x)}$, *D* is the molecular diffusion coefficient, and $r_i({{\mathbf {x}}},t)$ represents the space-time-dependent rate at which species *C* is produced, and species *A* and *B* are removed during the reaction. All chemical species are assumed to be non-sorbing species and to have the same diffusion coefficient. Initially, the resident water has concentration $c_0$ of reactant *B*. Then, water with concentration $c_0$ of species *A* is continuously injected into the water phase at the left domain boundary. As one water displaces the other, *A* and *B* diffuse across the interface, and the reaction proceeds to form the product *C*. The domain geometry, including the spatial distribution of the water and air phases shown in Fig. 1, is given by the experimental setup and data reported in Jiménez-Martínez et al. (2015).

The classical continuum approach is based on the longitudinal hydrodynamic dispersion coefficient $D^*$ and describes large-scale reactive transport in this setup by

3 $$\begin{aligned} \phi S_{w}\frac{\partial c_i({x},t)}{\partial t} + q \frac{\partial }{\partial x}c_i({x},t) - D^*\frac{\partial ^2}{\partial x^2} c_i({x},t) = r_i({x},t). \end{aligned}$$

where $\phi$ is the porosity, $S_{w}$ the water saturation, and *q* is the Darcy velocity in the water phase. The term $\phi S_{w}$ is the ratio of water phase in the domain and accounts for the presence of an immobile phase of air. The temporal evolution of the produced mass $m_C(t)$ under the assumption of remote boundary conditions at both ends of the experimental cell is given by Gramling et al. (2002)

4 $$\begin{aligned} m_C(t) = c_0 w \phi S_w \sqrt{\frac{4D^*t}{\pi }}, \end{aligned}$$

where *w* is the width of the domain.

We study reactive mixing in this setup in three complementary ways. The first is post-processing the experimental data for the transport of a conservative solute. This method is based on the fact that the reactive transport problem posed above can be fully described in terms of conservative components. This allows one to infer the mass production rate of *C* directly from the experimental data. The second method is fully numerical and solves the Stokes equation for pore-scale flow in the experimentally given distribution of the water phase. The reactive transport problem is then solved using a reactive random particle tracking method. The third method uses an upscaling model, the dispersive lamella approach, in order to predict the mass production from suitably defined effective dispersion coefficients. All three methods are outlined in the following.

### Experimental Data

#### Experimental Setup

In the following, we briefly describe the two experimental setups and data considered (Fig. 1). Both setups consider flow cells with quasi 2-D media composed of randomly located grains of varying diameter. The wetting fluid is a 60-40 % by weight water-glycerol solution with dynamic viscosity $\mu = 3.72 \times 10^{-2}$ kg m $\hbox {s}^{-1}$ and density $\rho = 1.099 \times 10^3$ kg $\hbox {m}^{-3}$. Air is used as non-wetting phase. After creating the spatially homogeneous phase distributions, a solution dyed with fluorescein is injected at constant flow rate $Q = 5.5\times 10^{-10}$ $\hbox {m}^3$ $\hbox {s}^{-1}$. Note that such a small flow rate does not displace the gas phase, that is, capillary forces are larger than viscous forces (Tang et al. 2019). $1.03 ^{-2}$ Time series of the experimental images did not show any displacement of the non-wetting phase.

For the first setup, the medium has dimensions of 130 mm of length and 82 mm of width, with thickness *h* = 0.5 mm. The medium is characterized by two length scales: the average throat width *a* = 1.07 mm and the average pore length $\ell _p$ = 1.75 mm. The porosity and absolute permeability are $\phi = 0.77$ and $\kappa = 7.5 \times 10^3$ $\hbox {mm}^2$, respectively. The saturation of the wetting phase, which is defined as the fraction of the total pore volume occupied by clusters of the wetting fluid, is $S_w = 0.70$. This scenario is denoted by SW70 in the following. It is used for the validation of the numerical flow and reactive transport model, and the dispersive lamella approach described in Sects. 2.2 and 2.3.

For the second experimental setup, the medium is composed of heterogeneously distributed circular grains of varying diameter which result in a porosity $\phi = 0.71$. The mean grain diameter is $d = 0.83$ mm and the average pore throat $a = 1.17$ mm and pore length $\ell _p = 1.85$ mm (Jiménez-Martínez et al. 2017). The dimensions of the medium are $l\times w = 132 \times 87$ $\hbox {mm}^2$ (in the *x*- and *y*-directions). We investigate three different water saturations $S_w = 0.71, 0.77, 0.83$. These scenarios are denoted by SW71, SW77 and SW83 in the following. Note that the ratio of water in the domain is the porosity, equal for the three scenarios, times the water saturation of the porous phase $\phi S_w$. A decrease in saturation generally leads to a modification of the flow paths, which results in a more heterogeneous velocity field (Leontidis et al. 2020; Velásquez-Parra et al. 2021). The mixing behaviors in these scenarios are investigated using the numerical simulations and the dispersive lamella approach validated for scenario SW70.

#### Reactive Mixing from Experimental Conservative Concentration Data

We use the experimental data of the conservative transport that occurs within the liquid phase to estimate the product formation from a fast reaction. Initially, the reactants A and B are segregated along a line interface normal to the flow direction at equal initial concentration $c_0$. We define the components $c_{AC}(\mathbf {x},t)=c_{A}(\mathbf {x},t)+c_{C}(\mathbf {x},t)$ and $c_{BC}(\mathbf {x},t)=c_{B}(\mathbf {x},t)+c_{C}(\mathbf {x},t)$, which by definition are conservative. The diffusion coefficient is assumed to be the same for all species.

For an instantaneous reaction, the reaction product can be calculated directly from the concentration $c_{AC}$ and $c_{BC}$ of the conservative species (Gramling et al. 2002; Perez et al. 2020) because species *A* and *B* cannot coexist. We identify $c_{AC}$ with the distribution of the conservative tracer from the laboratory experiments described above. The component $c_{BC}$, which is displaced by the inflowing reactant *A*, can be expressed as

5 $$\begin{aligned} c_{BC}(\mathbf {x},t) = 1 - c_{AC}(\mathbf {x},t). \end{aligned}$$

As *A* and *B* cannot coexist, the concentration of the reaction product *C* is simply given by

6 $$\begin{aligned} c_C(\mathbf {x},t) = \text {min}\left[ c_{AC}(\mathbf {x},t), c_{BC}(\mathbf {x},t)\right] . \end{aligned}$$

This expression predicts that $c_C$ will be produced within the mixing interface of reactants *A* and *B*, and its concentration peak will remain equal to $c_{0}/2$. The total mass of *C*, $m_C(t)$, can be found by integrating $c_C(\mathbf {x},t)$ over the whole domain:

7 $$\begin{aligned} m_C(t) = \int d\mathbf {x} c_C(\mathbf {x},t). \end{aligned}$$

### Numerical Simulations

#### Flow Within the Liquid Phase

The scenario SW70 (top left panel of Fig. 1) is used for model validation. It is represented in a regular grid consisting of 2370$\times$1504 pixels (corresponding to *x* and *y* dimensions, respectively), which yields a pixel resolution of 0.05 mm. The three other saturation degrees (${S_w}= 0.83, 0.77$, and 0.71) have dimensions of 3222$\times$2164 pixels with a resolution of 0.04 mm per pixel (see Fig. 1).

We calculate the flow field in the wetting phase by solving the continuity equation together with the steady state Stokes flow equation:

8 $$\begin{aligned}&\nabla \cdot \mathbf {v} = 0, \end{aligned}$$

9 $$\begin{aligned}&-\nu \nabla ^2 \mathbf {v} + \mathbf {v} \frac{\mathbf {\nu }}{k} = -\frac{1}{\rho }\nabla P. \end{aligned}$$

where $\mathbf {v}$ is the velocity vector (m $\hbox {s}^{-1}$) and *P* (kg $\hbox {m}^{-1}$ $\hbox {s}^{-2}$) is the pressure. The kinematic viscosity is determined from ratio of the viscosity $\mu$ and density $\rho$ of the water-glycerol solution and is given by $\nu = 3.4 \times 10^{-5} \hbox {m}^2 \hbox {s}^{-1}$. The term *k* on the left side of (9), where $k = h^2/12$, accounts for the constraint imposed on the flow by the gap (*h*) between top and bottom plates in the quasi 2-D experimental geometry following the methodology used in Horgue et al. (2013). This approximation provides accurate results at low Reynolds numbers (Ferrari et al. 2015).

Constant flow rate $Q = 5.5\times 10^{-10} \text {m}^3\text {s}^{-1}$ and constant pressure *P* = 0 kg $\hbox {m}^{-1}\hbox {s}^{-2}$ are imposed at the inlet and outlet of the domain, respectively. Solid obstacles and non-wetting phase are considered immobile and incompressible. No-slip condition is implemented at the water-air and water-solid interfaces. This assumption is valid because of the low flow rate *Q* used, which also prevents the deformation and displacement of air bubbles. Guédon et al. (2019) found no differences in the velocity distributions for slip and no-slip boundary conditions between wetting and non-wetting phases. Triadis et al. (2019) found the transport behavior is the same for slip and no-slip boundary conditions between water and air phase. The no-slip condition reduces significantly the computational cost (Guédon et al. 2019; Jiménez-Martínez et al. 2020).

**Fig. 2:**
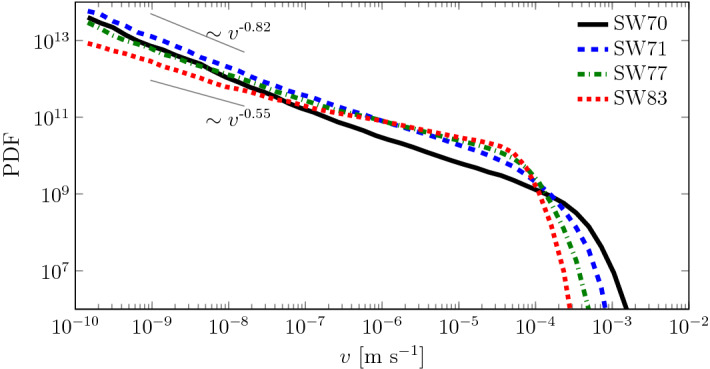
Probability density function (PDF) of velocity distributions for the validation geometry SW70 with $S_w = 0.7$ (black solid line), SW71 with $S_w = 0.71$ (blue dashed line), SW77 with $S_w = 0.77$ (green dash-dotted line), and SW83 with $S_w = 0.83$ (red dotted line)

Equations (8) and (9) are solved using the incompressible flow solver simpleFOAM from the open-source code OpenFOAM (Weller et al. 1998) modified to account for the $(\mu /k) \mathbf {v}$ term. Figure 2 displays the probability density function (PDF) $p_v(v)$ of velocity magnitudes within the fluid phase for the four saturation degrees shown in Fig. 1. All four are characterized by a power-law behavior of the form $p_v(v) \sim v^{\alpha -1}$ with $\alpha < 1$ at small velocities. The exponent $\alpha$ decreases with decreasing saturation (e.g., $\alpha = 0.45$ for $S_w=0.83$ and $\alpha = 0.18$ for $S_w = 0.7$), while the maximum velocity increases because the heterogeneity of the medium grows with decreasing saturation, thus creation preferential channel of high velocities. Velocity PDFs that exhibit small slopes at low velocities are known to trigger anomalous transport. The slope of the velocity PDF can be related to transport properties such as breakthrough curves: a smaller exponent of the slope results in a more anomalous transport (Puyguiraud et al. 2021).

#### Reactive Random Walk Particle Tracking Model

We analyze transport and mixing using numerical RWPT simulations combined with a probabilistic rule for the reaction. The advective-diffusive motion of reactant and product species particles is governed by the discretized Langevin equation (Risken 1996)

10 $$\begin{aligned} \mathbf {x}(t+\varDelta t) = \mathbf {x}(t) + \mathbf {v}[\mathbf {x}(t)]\varDelta t + \sqrt{2D\varDelta t}\varvec{\xi }(t), \end{aligned}$$

where $\mathbf {x}(t)$ is the position of the particle at time *t*. The advective step during a time interval $\varDelta t$ is based on a extension of the Pollock algorithm (Pollock 1988; Puyguiraud et al. 2019, 2020) that accounts for the no-slip conditions at non-wetting phase and solid grains. The $\varvec{\xi }(t)$ are independent identically distributed random variables that model particle motion due to diffusion. They are distributed according to a uniform distribution with mean 0 and unit variance in order to save the computational cost of generating Gaussian random numbers (Puyguiraud et al. 2020). The central limit theorem ensures that the sum of these increments is Gaussian. Particle trajectories are simulated until they exit the medium or react.

The transport scenarios are characterized by the Péclet number $Pe = a^2 \bar{v}/(2D\ell _p)$, which is the ratio of the characteristic diffusion time $\tau _D = a^2/(2D)$ across the average pore throat *a* and the advective time $\tau _v = \ell _p/\bar{v}$ over the average pore length $\ell _p$, where ${\bar{v}}$ is the average streamwise velocity. The diffusion coefficient is $D=1.5 \times 10^{-10}$ $\hbox {m}^2$ s$^{-1}$. The Péclet number is set for each scenario by scaling the velocity field to the needed value of $\bar{v}$.

The reactive transport simulation in the SW70 scenario uses a total of $N^0_{B} = 2\times 10^6$ particles to represent reactant *B*, and initial conditions are based on the experimental setup which can be expressed as a sharp interface between the two reactants. In the simulations, initially, the *B* particles are uniformly distributed in the pore space of the geometry between $x/\ell _p = 1$ and $x/\ell _p = 60$. We use this initial distribution of species *B* to ensure well-mixed conditions of reactants while reducing computational cost. Simulations in SW71 use $N^0_{B} = 3.65 \times 10^6$ particles, while $N^0_{B} = 2 \times 10^6$ particles are used in SW77 and SW83. The reactant *A* is introduced at the inlet using a flux-weighted injection, which is equivalent to a constant concentration boundary, and is continuously injected throughout the simulation. The variance of the straight initial interface between *A* and *B* particles is set equal to the initial interface width $\sigma _0^2$ recorded in the laboratory experiments (Jiménez-Martínez et al. 2015) in SW70.

Reactions are simulated following the reactive RWPT methodology presented in Perez et al. (2019a) for a sharp initial interface between reacting species. Here, we describe the main lines of the method. At each time step $\varDelta t$, we record the position of all particles. If an *A* particle is located at a distance *d* of a *B* particle that is smaller than the reaction radius $r = \sqrt{24D\varDelta t}$, they react with certainty, which represents the instantaneous kinetics of the irreversible bimolecular reaction. The limits and criteria for the choice of the reaction radius for finite kinetics can be found in Perez et al. (2019a). The time step $\varDelta t$ here is chosen such that *r* is smaller than the pixel size. This choice ensures that reactions at solid boundaries are correctly accounted for; specifically, it ensures that particles separated by a solid pixel cannot not react. For SW70, we set $\varDelta t = 0.1$ s, which gives $r = 1.6 \times 10^{-5}$, while the pixel size is $5\times 10^{-5}$ m. For SW71, SW77 and SW083 we set $\Delta t = 0.05$ s, which gives $r = 1.35 ^{-5}$ m with a pixel size of $4 ^{-5}$ m.

After reaction, the *A* and *B* particles are removed and a particle *C* is placed at the middle point between the locations of the *A* and *B* particles. The migration of *C* particles in the domain also follows the transport rules specified in Eq. (10).

We also consider conservative transport scenarios to probe the dispersion of the mixing interface. They are characterized by an instantaneous line source perpendicular to the mean flow direction located at $x=1$ and composed of 1504 point injections (an injection in each pixel at $x=1$), in the validation case SW70 (Fig. 1, top left panel), and 2164 point injections other three saturation cases (Fig. 1, remaining panels). At each point of the line source, 5000 particles are released to capture the impact of heterogeneity and diffusion on the mixing interface (see Sect. 2.3).

### The Dispersive Lamella Approach

We use the dispersive lamella approach to predict mixing and reaction under different degrees of saturation. It has been previously employed to describe reactive mixing in Poiseuille flow (Perez et al. 2019a) and in two-dimensional saturated porous media (Perez et al. 2020; Puyguiraud et al. 2020). This approach decomposes the mixing interface into partial plumes originating from point-like injections that constitute the initial particle distribution. The dispersive lamella approach approximates the concentration distributions associated to these partial plumes, that is, the transport Green function, by a Gaussian distribution that is characterized by an effective dispersion coefficient. In this framework, the total product mass is given by (Perez et al. 2019a, 2020; Puyguiraud et al. 2020)

11 $$\begin{aligned} m_C(t) = c_0 w \phi S_w \sqrt{\frac{2\sigma _e^2(t)}{\pi }}, \end{aligned}$$

where *w* the width of the medium and $\sigma _e^2(t)$ is the effective variance of the mixing interface. The latter is the key quantity in this approach and is discussed in more detail in the following.

#### Effective Variance

To obtain an expression for the effective variance, we first define the moments and the spatial variance of the transport Green function $g({{\mathbf {x}}},t|y)$,

12 $$\begin{aligned} m_i(t| y') = \int d\mathbf {x}\, x^i g(\mathbf {x},t| y'), \,\,\,\,\, \sigma ^2(t| y') = m_2(t| y') - m_1(t| y')^2. \end{aligned}$$

The first moment $m_1(t|y)$ denotes the center of mass position of the point-like plume that originates from *y*. The transport Green function $g({{\mathbf {x}}},t|y')$ satisfies

13 $$\begin{aligned} \frac{\partial g(\mathbf {x},t| y')}{\partial t} + \mathbf {v(x)}\cdot \nabla g(\mathbf {x},t| y') - D \nabla ^2 g(\mathbf {x},t| y') = 0, \end{aligned}$$

for the initial condition $g({{\mathbf {x}}},t=0|y') = \delta (x) \delta (y - y')$. The concentration $c({{\mathbf {x}}},t)$ can be expressed in terms of the Green function by integration over the initial solute distribution, which here is given by the line source. The initial concentration distribution along a line source can be written as

14 $$\begin{aligned} c({{\mathbf {x}}},t = 0) = \frac{1}{w \phi S_{w}} \delta (x) \rho (y), \end{aligned}$$

where $\rho (y)$ equals 1 if $y$ is in the pore space (water saturated phase) and 0 else. Thus, $c({{\mathbf {x}}},t)$ can be written as

15 $$\begin{aligned} c(\mathbf {x},t) = \frac{1}{w\phi S_{w}}\int _0^w dy' \rho (y')g(\mathbf {x},t| y'). \end{aligned}$$

Using these definitions, the moments and variance of the $c({{\mathbf {x}}},t)$ can be expressed by

16 $$\begin{aligned} m_i(t) = \frac{1}{w\phi S_{w}} \int _0^w dy' \rho (y') m_i(t|y'). \end{aligned}$$

The mixing volume is measured by the effective variance, which is defined by the average of $\sigma ^2(t|y)$ over all source positions

17 $$\begin{aligned} \sigma ^2_e(t) = \frac{1}{w\phi S_{w}} \int _0^w dy' \rho (y') \sigma ^2(t|y'). \end{aligned}$$

It denotes the average variance of the mixing interface that is formed by the ensemble partial plumes that originate from the line source at $x = 0$. The effective variance is determined from both the experimental conservative tracer data and the numerical flow and transport simulations as outlined in the following.

#### Effective Variance from Experimental Data

It is not possible to discriminate partial plumes that are equivalent to the transport Green function from the experimental concentration images. However, as the aim is to characterize the variance of the mixing interface, we define the effective variance in terms of the moments of the mixing function (Perez et al. 2020)

18 $$\begin{aligned} \varTheta (\mathbf {x},t) = c_{AC}(\mathbf {x},t)[1-c_{AC}(\mathbf {x},t)]. \end{aligned}$$

This quantity is linked to the segregation intensity (Danckwerts 1952). It tends to zero away from the mixing zone and thus delineates the mixing interface between *A* and *B*. The longitudinal moments of $\varTheta (\mathbf {x},t)$ are defined by

19 $$\begin{aligned} \mu _i(t|y) = \int dx \, x^i \varTheta (\mathbf {x},t). \end{aligned}$$

The width of the interface at a position $y$ is evaluated in terms of the horizontal variance

20 $$\begin{aligned} \kappa (y,t) = \frac{\mu _2(t|y)}{\mu _0(t|y)} - \frac{\mu _1(t|y)^2}{\mu _0(t|y)^2}. \end{aligned}$$

We find the effective variance $\kappa _e^2(t)$ of $\varTheta ({{\mathbf {x}}},t)$ by vertical averaging

21 $$\begin{aligned} \kappa _e^2(t) = \int _0^w dy \, \kappa (t|y). \end{aligned}$$

The effective variance $\sigma _e^2(t)$ of the concentration distribution $c_{AC}({{\mathbf {x}}},t)$ is related to $\kappa _e(t)$ by Perez et al. (2020)

22 $$\begin{aligned} \sigma _e^2(t) = \frac{6}{5} \kappa _e(t), \end{aligned}$$

Note that the factor 6/5 is the multiplier between the spatial variance of $c$ and the normalized auxiliary function $c(1-c)$ for a normalized Gaussian *c* (Perez et al. 2020).

#### Effective Variance from Numerical Simulations

In the numerical simulations, an ensemble of particles is released along a line source, which consists of $N_p$ injection points of size $\varDelta y$, equal to the pixel size, which mimic the transport Green’s functions. At each injection point $N_0$, particles are uniformly distributed within the pixel. The center of the point injection *i* is located at $(0, y_i)$ and the position of a particle *j* at time *t* that was released within this point injection is denoted by ${{\mathbf {x}}}_{(j)}(t|y_i)$. The moments $m_k(t|y_i)$, for each point injection *i*, are defined in terms of ${{\mathbf {x}}}_{(j)}(t|y_i)$ as

23 $$\begin{aligned} m_k(t|y_i) = \frac{1}{N_{0}} \sum \limits _{j = 1}^{N_{0}} x_{(j)}^{k}(t|y_i), \end{aligned}$$

where $N_{0}$ is the number of particles that are released in each point injection $i$ and that are still in the system after time $t$. The variance of a point injection is simply defined as

24 $$\begin{aligned} \sigma ^2(t|y_i) = m_2(t|y_i)-m_1(t|y_i)^2. \end{aligned}$$

The effective variance is calculated as the average of all the point injection variances:

25 $$\begin{aligned} \sigma _e^2(t) = \frac{1}{N_p} \sum \limits _{i = 1}^{N_p} \sigma ^2(t|y_i), \end{aligned}$$

where $N_p$ is the number of release points in the initial line.

## Results

In this section, we study the dynamics of mixing under different saturation degrees. To this end, we first validate the numerical flow and transport simulations and the dispersive lamella approach with the experimental data from scenario SW70, which then are used to analyze the impact of saturation for the scenarios SW71, SW77, and SW83 on reactive mixing.

Reactions are considered instantaneous, which means that the characteristic reaction time is much smaller than the characteristic advection time. The Péclet number here is $Pe = 78$ for scenario SW70, equal to the $Pe$ in Jiménez-Martínez et al. (2015), and $Pe = 70$ for scenarios SW71, Sw77 and SW83. This means transport is advection dominated in all of them.

In the following space is non-dimensionalized by $\ell _p$, the average pore length, and time by $t_f = V_0 \phi S_{w}/Q$, where $V_0$ is the volume of the flow cell. The characteristic time $t_f$ corresponds to the time at which the volume of injected fluid equals the pore volume occupied initially by the resident fluid. Dimensionless time is denoted by pore volumes $PV = t/t_f$. Note that the fluid volume in the system differs between geometries and between saturations for the same geometry. This means that for the different scenarios the same fluid volume is injected at different PV.

### Validation by Experimental Data

**Fig. 3:**
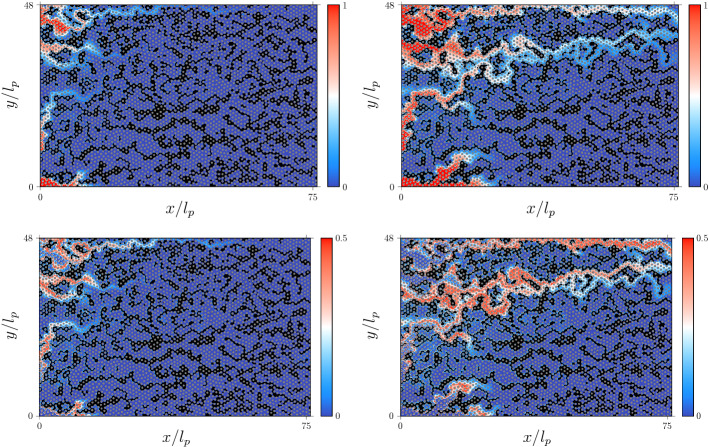
Concentration maps of species *A* (top left and right panels) and product *C* (bottom left and right panels) at $PV = 0.0714$ (left column) and $PV = 0.4281$ (right column) pore volumes for scenario SW70

Figure 3 shows the distribution of reactant $A$ and product $C$ at $PV = 0.0714$ and $PV = 0.4281$ for the validation scenario SW70. At early times, the invasion of *A* in the porous medium leads to a finger structure (Fig. 3, top left panel). Preferential flow paths further stretch the interface (Fig. 3, bottom left panel). As a consequence, the distribution $c_C(\mathbf {x},t)$ of the reaction product $C$ is highly heterogeneous and essentially localized in the preferential flow paths (Fig. 3, right panels).

The RWPT model is validated by comparing the evolution of the mixing interface in terms of the effective variance, and the predicted mass $m_{C}$ of reaction product to the values estimated from experimental data using the methodology detailed in Sect. 2.1. The dispersive lamella approach is validated by comparison of the predicted product mass to the product mass estimated from the experimental data.

**Fig. 4:**
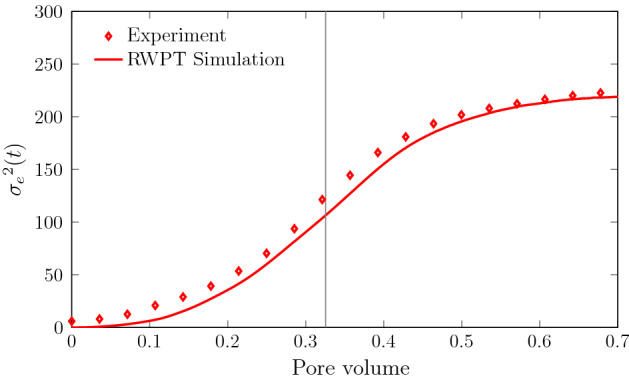
Evolution of effective variance $\sigma _e^2(t)$ from experimental data (symbols) and the RWPT simulation (solid lines) for the scenario SW70. The gray solid line denotes the time at which the plume reaches the outlet

Figure 4 shows the evolution of the effective variance computed from experimental data and from the RWPT simulations. $\sigma _e^2(t)$ increases with time due to diffusion and velocity variability along the cross section of the medium. The mismatch between the numerical and experimental data for $\sigma _e^2(t)$ at early times is due to the fact that the numerical simulations consider a straight interface at $t=0$, while the actual initial interface of the experiment is spread out.

The effective variance evolves due to diffusion and deformation of the interface, but it is not directly affected by the formation of fingers, and thus evolves at a slower rate. Recall that $\sigma _e^2(t)$ quantifies the effective width of the mixing front. At late times, its evolution slows down, on the one hand, because the behavior is not ballistic any longer and, on the other hand, because the interface approaches the end of the flow cell. Thus, the growth rate slows down almost to a plateau as the more advanced interface features leave the domain. We mark these finite size features in our RWPT simulations at a time when the first particles $N(t)/N_0 = 10^{-3}$ reach the outlet, which is represented with a gray line in Fig. 4. The numerical random walk particle tracking simulations capture the full evolution of the effective variance, except for the very early times, as discussed above.

**Fig. 5:**
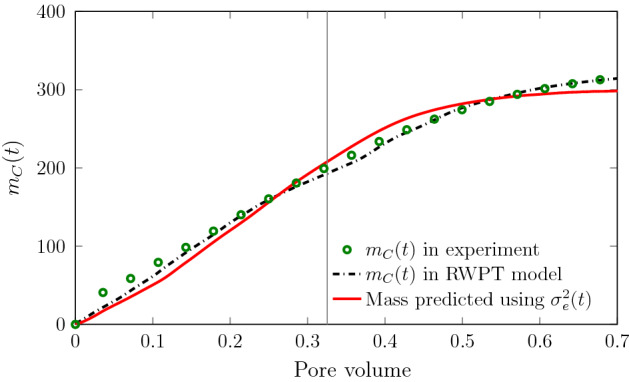
Evolution of product mass $m_C(t)$ for scenario SW70 from the experimental data (green symbols), the results obtained from the reactive RWPT simulations (black dash-dotted line), and the predictions from the dispersive lamella (11) parameterized by $\sigma _e^2(t)$ (red solid line). The gray solid line denotes the time at which the plume reaches the outlet

Figure 5 shows the evolution of $m_C(t)$ estimated from the experimental data, the RWPT simulation, and the dispersive lamella approach. We find good agreement between experimental and reactive RWPT results, which validates the numerical flow and transport model. The fast increase in $m_C(t)$ suggests that the majority of *C* is being formed in the preferential channels. The dispersive lamella prediction captures well the preferential reaction mechanisms occurring in the fast channels and in particular describes well the strong nonlinear increase in the product mass at short and intermediate times due to the combined action of advective heterogeneity and transverse mixing. This evolution of the product mass cannot be explained in terms of constant hydrodynamic dispersion coefficients, which predicts an increase as $\sqrt{t}$.

### Impact of Saturation on Dispersion and Reactive Mixing

We quantify the impact of saturation degree and flow heterogeneity on dispersion and reactive mixing using direct numerical simulations in the saturation scenarios SW71, SW77 and SW83 (Jiménez-Martínez et al. 2017). These scenarios are all characterized by $Pe = 70$.

#### Dispersion of the Mixing Interface

**Fig. 6:**
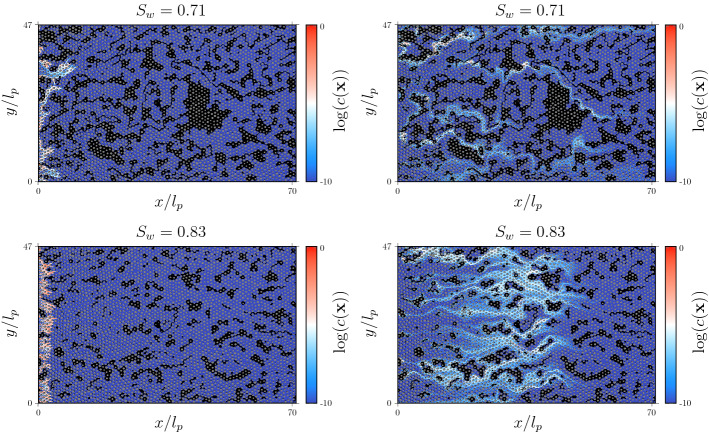
Map of the logarithm of the concentration of a conservative solute at $PV = 0.049$ (left column) and $PV = 0.82$ (right column) pore volumes for scenarios (left) SW71 and (right) SW83

Figure 6 illustrates the concentration distribution evolving from a line source at $PV = 0.049$ and $PV = 0.82$ in the SW71 and SW83 scenarios to show the initial deformation at early times, and the transition to finite size effects in SW71 while in SW83 is not observed yet. The deformation of the line is analogous to the deformation of the mixing interface between the $A$ and $A$ species. The concentration fields $c(\mathbf {x},t)$ formed by the particles in the RWPT model are computed using an adaptive Gaussian kernel density estimator (Botev et al. 2010) to minimize the fluctuations involved in the reconstruction of concentrations maps due to high deformation of the particle plume. At early times, the interface is strongly deformed due to medium and flow heterogeneity. This effect is stronger in the SW71 than in the SW83 scenario due to stronger heterogeneity. This causes a fast growth of the interface length and width, which enhances the mixing and reaction among the initially segregated reactants. With increasing time, the fast channels in the SW71 scenario stretch the interface, which leads to strong concentration gradients. The arrival of solute in the fast channels at the outlet marks the time after which finite size effects become important as observed in Figs. 4 and 5 for the spatial variances and product mass evolution in scenario SW70.

**Fig. 7:**
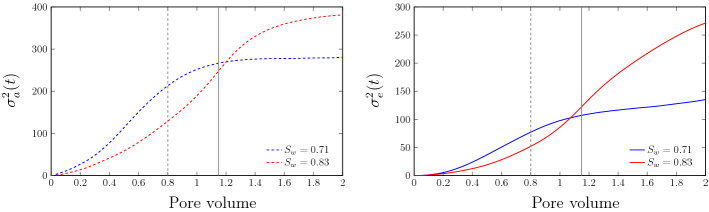
Evolution of effective ($\sigma _e^2(t)$) variances from RWPT simulations for saturation $S_w$ = 0.71 (blue line) and $S_w$ = 0.83 (red line). The gray dashed and solid lines denote the time after which the first particles $N(t)/N_0 =10^{-3}$ reach the outlet for scenarios SW71 and SW83, respectively. Finite size effects start to emerge at that time

Figure 7 shows the evolution of the effective variances for scenarios SW71 and SW83. We observe that $\sigma _e^2(t)$ grows faster for scenario SW71 than for SW83, which is due to the stronger local deformation of the partial plumes that constitute the line source. This implies that the mixing volume increases faster in scenario SW71 than in SW83. More saturated porous media exhibit a less heterogeneous velocity field (Triadis et al. 2019; Velásquez-Parra et al. 2021) and therefore less deformation. This is also manifest in the enhanced spreading of the interface (Boon et al. 2017; Comolli et al. 2019; Hidalgo et al. 2021) shown in Fig. 6. After the solute breaks through at the outflow boundary (indicated by the vertical lines in Fig. 7), the effective variance increases to larger values for scenario SW83 than for SW71. This can be attributed to the fact that the interface growth for SW71 is dominated by a few fast channels, while it is more dispersive, or equally distributed between more smaller features for SW83, see also Fig. 6.

Our analysis of $\sigma _e^2(t)$ suggests that less water saturation leads to a faster growth of the interface which is attributed to higher interface distortion caused by the velocity fluctuations associated with the formation of preferential paths of high velocity, and stagnation zones.

#### Reactive Mixing

**Fig. 8:**
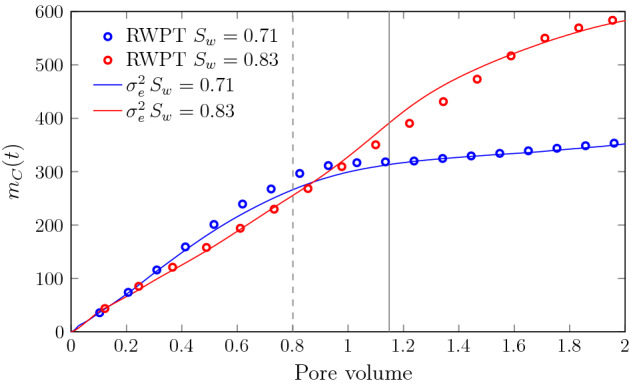
Evolution of product mass $m_C(t)$ from the RWPT model (green symbols), and the dispersive lamella prediction for scenarios (blue) SW71 and (red) SW83. The gray dashed and solid lines denotes the time after which the first particles $N(t)/N_0 =10^{-3}$ reach the outlet for scenario SW71 and SW83, respectively. Finite size effects start to emerge at that time

Figure 8 shows the evolution of the total mass of product $C$ for scenarios SW70 and SW83 from the reactive RWPT simulations and the dispersive lamella model estimate. The global reaction behavior in terms of $m_C(t)$ for scenario SW77 (not shown) is qualitatively similar to the results presented in Fig. 8, but the reaction plateau caused by finite medium size effects occurs later than for scenario SW71 but earlier than for SW83.

The flow heterogeneities due to preferential channels induce a deformation of the interface that enhances the reaction at early times compared to the behavior expected from molecular diffusion only. Scenario SW71, which has the lowest saturation and highest heterogeneity among the scenarios under consideration, shows a higher reactivity than scenario SW83 characterized by a faster production of $C$. Note that for scenarios SW71 and SW83, the same PV corresponds also to the same mass of $A$ injected. After the breakthrough of the interface at the outflow boundary, the mass production slows down in both scenarios, with a stronger effect on scenario SW71 than SW83. As discussed above, the mixing interface for SW71 is dominated by a few fast features, while the interface is more dispersive for scenario SW83. Thus, scenario SW83 continues producing $C$ after the interface breaks through, while for scenario SW71 the production of $C$ is almost stagnant. The production $C$ mirrors the evolution of the effective variance discussed in the previous section. Thus, the dispersive lamella model, which is parameterized in terms of $\sigma _e^2(t)$, captures the evolution of $m_C(t)$ in both saturation cases over the full time range.

**Fig. 9:**
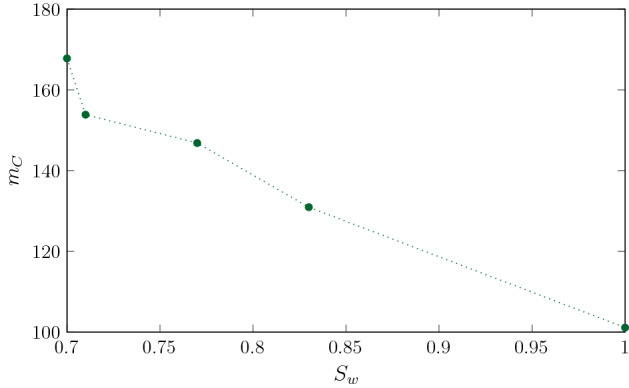
Total mass of $C$ formed for scenarios: SW70 at $PV = 0.24$, SW71 at $PV = 0.4$, SW77 and SW83 at $PV = 0.31$ in $S_w = 1$ which are the pore volumes at which the same amount of reactant $A$ is injected in all scenarios

Figure 9 shows the global impact of saturation on $m_C$ when the same dimensionless mass $m_A = 271$ of reactant *A* has been injected in all the geometries studied. This time corresponds to $PV = 0.24$ in SW70 and $PV = 0.4$ in SW71, SW77 and SW83. Note that, for reference, we also plot the results of the total mass of product $C$ for the fully saturated case $S_w = 1$ ($Pe = 54$) that was analyzed in Perez et al. (2020). Strong flow heterogeneities in low saturation systems lead to strong deformation of the mixing interface and subsequently to an increase in the mixing width compared with higher saturation degrees. These findings emphasize that mixing and reactions are enhanced as the degree of saturation decreases and the heterogeneity of the flow field increases.

## Conclusions

We quantify the impact of water saturation on dispersion and mixing in heterogeneous two-dimensional porous media using a combination of experimental data, numerical flow and transport simulations, and the dispersive lamella method as an upscaled mixing model. Experimental conservative transport data are used to estimate the evolution of the mixing interface in terms of the effective width of the mixing interface. Reactive mixing is estimated from the experimental data based on an exact algebraic map between conservative and reactive species for an instantaneous irreversible bimolecular reaction. The numerical simulations solve Stokes flow in the water phase in the pore space, transport and reaction are solved by reactive random walk particle tracking. The numerical model is validated by comparison with the effective variance and the reactive mixing results estimated from the experimental data for scenario SW70. Dispersion and reactive mixing for the saturation scenarios SW71, SW77 and SW83 are then analyzed using the numerical flow and transport simulations. The upscaling of the observed reaction behaviors is studied with the dispersive lamella approach. Solute mixing is quantified by the effective variance of the concentration distribution evolving from an initial line source in the different medium and saturation scenarios. The deformation and mixing of the line quantifies the spreading and mixing of the mixing interface between the initially segregated solutions of $A$ and $B$. Our analysis shows that mixing is enhanced with decreasing saturation which suggests that mixing and reaction is dependent on saturation degree. This is traced back to the increase in flow heterogeneity and the creation of preferential flow channels. The same heterogeneity mechanisms lead to increased chemical reactions as saturation decreases due to greater distortion of the mixing interface that results in a greater mixing area. The effective reaction rate, measured by the evolution of product mass, is correctly quantified by the dispersive lamella method in terms of saturation-dependent effective dispersion coefficients.

Finite size effects limit the growth of the interface. This is more pronounced for low saturations, for which the interface is dominated by a few fingers created by fast transport in preferential channels. We observe that the influence of stagnant zones, which become more frequent with decreasing saturation, plays only a minor role for mixing and reaction, which are concentrated in the fast channels. For small $Pe$, we expect similar mixing and reaction behaviors in the different geometries, see also Alhashmi et al. (2016). The large-scale reactive mixing behavior is captured by the dispersive lamella approach that quantifies the evolution of the product mass in terms of the (non-Fickian) evolution of the effective variance. Note that the porosity in our study is relatively high in our study compared to soils or aquifer rocks. However, the results presented here are expected to be valid also at smaller porosity as long as a connected fluid phase exists. Furthermore, the assumption underlying the dispersive lamella method is that the Green function averaged along the interface can be represented by a Gaussian. This assumption needs to be scrutinized for different types of porous media.

These results and the underlying mechanisms can be generalized to three-dimensional systems where a deformed reaction front leading to enhanced reaction dynamics is expected due to the presence of helical flow components and transverse mixing. However, the strong changes in structure and velocity heterogeneity observed in a narrow saturation range are most likely specific to two dimensions. Structural changes are more drastic in two than in three spatial dimensions because connectivity is higher in three dimensions.

Our findings elucidate the favorable effect of partial saturation on solute mixing and chemical reactions in the Earth’s critical zone and emphasize the potential of unsaturated media as efficient chemical reactors despite the lower fluid volume compared to saturated media. The key mechanisms are related to the strong increase in the heterogeneity of the fluid phase with decreasing saturation.
